# Evaluation of Essential, Toxic and Potentially Toxic Elements in Leafy Vegetables Grown in the Canary Islands

**DOI:** 10.3390/toxics11050442

**Published:** 2023-05-07

**Authors:** Verónica Martín-León, Carmen Rubio, Ángel Rodríguez-Hernández, Manuel Zumbado, Andrea Acosta-Dacal, Luis Alberto Henríquez-Hernández, Luis D. Boada, María del Mar Travieso-Aja, Octavio P. Luzardo

**Affiliations:** 1 Public Health Laboratory of Las Palmas, Canary Islands Government Health Service, 35004 Las Palmas de Gran Canaria, Spain; vemartinleon@hotmail.com; 2 Toxicology Department, Universidad de La Laguna, S/C de Tenerife, 38071 La Laguna, Spain; 3 Toxicology Unit, Research Institute of Biomedical and Health Sciences (IUIBS), Universidad de Las Palmas de Gran Canaria, Paseo Blas Cabrera s/n, 35016 Las Palmas de Gran Canaria, Spain; 4 Spanish Biomedical Research Center in Physiopathology of Obesity and Nutrition (CIBERObn), 28029 Madrid, Spain

**Keywords:** essential elements, potentially toxic elements (PTE), rare-earth elements (REE), minor elements (ME), heavy metals, lead, cadmium, mercury, arsenic, dietary exposure, toxicological risk

## Abstract

Forty-seven elements in leafy green vegetables were studied to estimate the daily intakes from this food category in different scenarios (average and high consumers) and age groups of the Canary Islands population. The contribution of the consumption of each type of vegetable to the reference intakes of essential, toxic and potentially toxic elements was assessed and the risk–benefit ratio was evaluated. The leafy vegetables that provide the highest levels of elements are spinach, arugula, watercress and chard. While spinach, chard, arugula, lettuce sprouts and watercress were the leafy vegetables with the highest concentrations of essential elements (38,743 ng/g of Fe in spinach, 3733 ng/g of Zn in watercress), the high levels of Mn in chard, spinach and watercress are noteworthy. Among the toxic elements, Cd is the element with the highest concentration, followed by As and Pb. The vegetable with the highest concentration of potentially toxic elements (Al, Ag, Be, Cr, Ni, Sr and V) is spinach. In average adult consumers, while the greatest contribution of essential elements comes from arugula, spinach and watercress, insignificant dietary intakes of potentially toxic metals are observed. Toxic metal intakes from the consumption of leafy vegetables in the Canary Islands do not show significant values, so the consumption of these foods does not pose a health risk. In conclusion, the consumption of leafy vegetables provides significant levels of some essential elements (Fe, Mn, Mo, Co and Se), but also of some potentially toxic elements (Al, Cr and Tl). A high consumer of leafy vegetables would see their daily nutritional needs regarding Fe, Mn, Mo, and Co covered, although they are also exposed to moderately worrying levels of Tl. To monitor the safety of dietary exposure to these metals, total diet studies on those elements with dietary exposures above the reference values derived from the consumption of this food category, mainly Tl, are recommended.

## 1. Introduction

Green leafy vegetables are common components of the human diet, as they are sources of fiber, vitamins and essential elements [1]. The latter (iron (Fe), zinc (Zn), copper (Cu), selenium (Se), manganese (Mn), molybdenum (Mo), and cobalt (Co)) are considered essential elements for life because an insufficient intake causes functional deficiencies, which are reversible if the element returns to adequate concentrations, and their effect cannot be replaced by any other element. Although these elements account for only 0.02% of the total body weight, they play an important role as they are essential for the maintenance of good health and the prevention of disease [2,3,4].

Nevertheless, the safety of leafy green vegetables is a concern, since these vegetables have been shown to accumulate toxic metals (cadmium (Cd), lead (Pb), arsenic (As) and mercury (Hg)) to a greater extent than other vegetables [5]. Thus, the consumption of leafy vegetables has been identified as a relevant source of human Cd [6,7,8,9,10], Pb [7,8,10,11,12,13], As [11], and Hg [10,12,14,15]. Previous dietary exposure studies [16,17] in the Canary Islands showed that total adult Cd and Pb dietary intakes were 0.16 µg/kg/day and 72.8 µg/day, respectively, below the reference intake limits set by EFSA and not posing a health risk for consumers.

In addition, leafy green vegetables may generate health risks due to their occurrence of other potentially toxic elements (PTE) such as Ag (silver), Al (aluminum), Ba (barium), Be (beryllium), Cr (chrome), Ni (nickel), Sb (antimony), Sn (tin), Sr (strontium), Tl (thallium), U (uranium), rare-earth elements (REEs) such as Ce (cerium), Dy (dysprosio), Er (erbium), Eu (europium), Ga (gallium), Gd (gadolinium), Ho (holmium), In (indium), La (lanthanum), Lu (lutetium), Nb (niobium), Pr (praseodymium), Sm (samarium), Ta (tantalum), Tb (terbium), Tm (thulium), Y (yttrium), Yb (yttervium) and minor elements (MEs) such as Au (gold), Bi (bismuth), Pt (platinum), Th (thorium), V (banadium). These PTE, REEs and MEs are of increasing concern as emerging environmental contaminants [18,19]. A study conducted by González-Weller et al. [20] estimated the total intake of Al by the Canary population (10 mg/day for a person weighing 70 kg), evaluating this exposure as lower than the tolerable weekly intake (1 mg/kg/week) established by the EFSA [21]. A recent study detected the presence of REEs in vegetables in China and leafy vegetables showed the highest concentrations. Nevertheless, the risk to human health from their consumption was estimated to be significantly lower than the reference intake values [22,23,24,25].

Multiple factors can increase the accumulation of toxic and potentially toxic elements in the environment and crops, such as the intensive use of agrochemicals, application of sewage sludge to croplands, atmospheric deposition [26,27], and electronic products discarded as part of the electronic waste (e-waste), among others. Between 60 and 64 elements from the periodic table are used in the manufacture of a cell phone, while a computer contains more than thirty elements. In general, almost 60% of the weight of an electronic device is due to the presence of metals [18]. Because of this, a list of 50 “essential elements” for the technology industry has recently been compiled, including the previously mentioned classical heavy metals and trace elements, as well as REEs, precious metals and other elements, some of which are extremely scarce in the earth’s crust [18]. Many of these elements are included in the list of priority pollutants prepared by the Agency for Toxic Substances and Disease Registry (ATSDR) [28].

These new “essential elements” for industry are now being studied in terms of their toxicity, as it has been observed that their presence in living beings has been increasing in recent years [19,23,24,25,29,30,31,32,33]. Their toxic actions include cell damage, interference with enzymes, proteins and macromolecules, damage to cell organelles, and even carcinogenesis, according to available epidemiological and experimental studies [34]. For the REEs group, different adverse health effects have been reported [35], such as anassociation with cell proliferation [36], alterations in estrogenic differentiation [37], the promotion of lipid peroxidation [38], formation of reactive oxygen species [39] and decrease in hemoglobin content [19].

In Spain, in addition to lettuce and spinach, which are the most consumed leafy green vegetables at the European level, there is a significant consumption of chard, which, within the EU, is almost exclusive to Spain [40]. Furthermore, within Spain, there are regions in which, due to culinary traditions, other green leafy vegetables are also consumed in extremely high quantities. This is the case for the Canary Islands, where large quantities of watercress (*Nasturtium officinale*) are consumed according to the ENCA, the nutritional survey for the Canary Islands [41]. In the Canary Islands, the consumption of chard and watercress is as high as that of spinach and lettuce [41,42,43].

The Canary Islands are one of the so-called outermost territories of the EU due to their remoteness from continental Europe. For this reason, highly perishable products such as green leafy vegetables are mostly produced in the region, and only a small amount of the total consumed quantity is imported from continental Spain [40].

As mentioned, multiple factors can increase this accumulation of essential, toxic and potentially toxic elements in leafy vegetables [26,27]. In the Canary Islands, there are several of these circumstances. On the one hand, this region has one of the highest uses of pesticides in Europe. According to calculations, in 2001, the Canary Islands used 12 times more pesticides per hectare than the rest of Spain [44], and the situation has not improved 20 years later [45]. Some previous studies [46] have already highlighted the interactions and correlations between heavy metals and pesticides in this food category. In addition to the application of agrochemicals or other practices that could increase the number of elements in the croplands, Saharan dust intrusion (called “calima”) occurs frequently throughout the year in this archipelago. Although few studies are available, it has been reported that Saharan dust, and general particulate matter (P10, P25), can carry large amounts of some elements [47,48], including REEs [19,48,49]. Therefore, a plausible hypothesis is that frequent “calima” arrivals to the Canary Islands could influence the content of all these elements in locally grown vegetables.

This novel study was designed to a) determine the levels of essential, toxic and potentially toxic elements in one food category (leafy green vegetables) grown in the Canary Islands, b) assess the dietary exposure to these elements from this food group, and c) estimate, for the Canary Islands population, the contribution and risk–benefit ratio of the consumption of each type of leafy vegetable according to the reference intakes of these essential, toxic and potentially toxic elements.

## 2. Materials and Methods

### 2.1. Sampling

**Figure 1:**
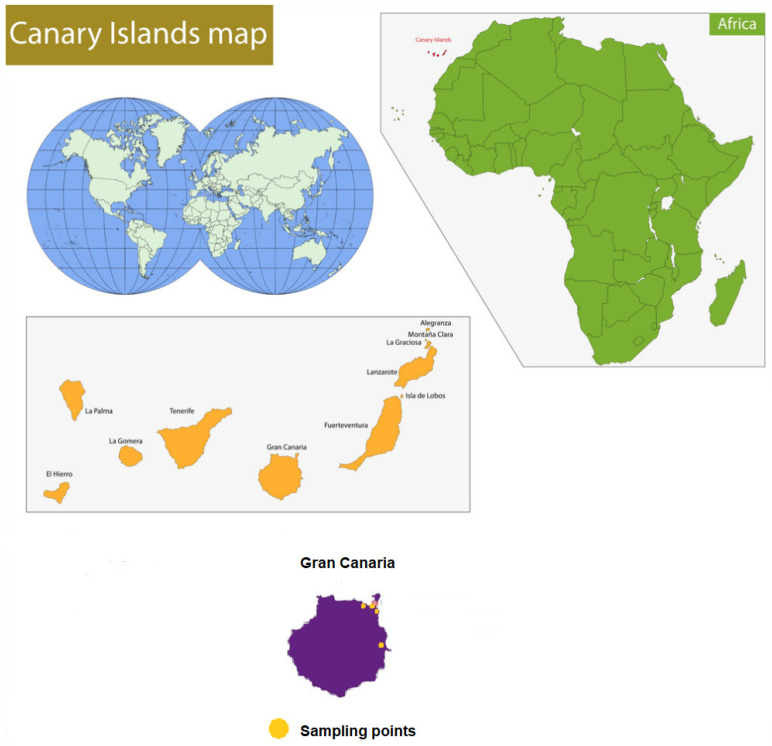
Canary Islands location and sampling points.

A total of 244 samples of leafy vegetables were studied. The samples were acquired from large supermarkets located on the island of Gran Canaria but representative of the whole archipelago market, since these points of sale are also located on the other seven islands (Figure 1). Sampling took place in two periods: from April 2016 to September 2016 (winter season) and from October 2016 to March 2017 (summer season).

Sampling considered the consumption patterns of the Canary Island population [41,43], and included 56 samples of watercress (*Nasturtium officinale*); 42 samples of chard (*Beta vulgaris*); 36 samples of romaine lettuce (*Latuca sativa. longitolia*); 22 samples of iceberg lettuce (*Latuca sativa*); 18 samples of baby lettuce; 16 samples of arugula (*Eruca vesicaria*); 30 samples of spinach (*Spinacia oleracea*); and 24 samples of lamb’s lettuce. Upon arrival at the laboratory, the samples were examined and cleaned of dirt with a damp paper towel, and inedible or damaged outer leaves were removed. After cleaning, the samples were frozen. The maximum storage period before analysis never exceeded six weeks.

### 2.2. Elements Analyzed and Analytical Procedure

Essential elements, toxic elements and a set of other elements, Agency for Toxic Substances and Disease Registry (US-ATSDR) priority list elements, REEs and other minority elements (ME) were studied. In total, 47 elements (Fe, Zn, Cu, Se, Mn, Mo, Co, Cd, Pb, As, iAs, Hg, MeHg, Ag, Al, Ba, Be, Cr, Ni, Sb, Sn, Sr, Tl, U, Ce, Dy, Er, Eu, Ga, Gd, Ho, In, La, Lu, Nb, Pr, Sm, Ta, Tb, Tm, Y, Yb, Au, Bi, Pt, Th, V) were investigated in leafy green vegetables.

All samples (ready-to-eat leafy vegetables) were ground and homogenized manually using a metal-free Teflon mortar until a homogeneous mass was formed. Three digests (extractions) were performed in parallel. Acid digestion of the homogenized samples was performed using a microwave digester (Ethos Up, Milestone SRL, Sorisole, Italy). For this purpose, 500 mg of vegetable homogenate was weighed (wet weight) into the digestion vessels and 50 μL of the internal standard solution (Sc, Ge, Rh and Ir were added at a stock concentration of 20 mg/mL each) (CPA Chem, Stara Zagora, Bulgaria), 2.5 mL of concentrated HNO_3_ (65%) (Merck KGaA, Darmstadt, Alemania) and 7.5 mL of Mili-Q water (Merck KGaA, Darmstadt, Alemania) were added to each sample. The digester was programmed in three stages at 1800 W of power: a first stage of 5 min at 100 °C, followed by a 5 min stage at 150 °C, followed by a third stage of 15 min at 200 °C. After cooling, the complete digests (extractions) were transferred to conical-bottom polypropylene tubes and diluted to 15 mL with Mili-Q water (Merck KGaA, Darmstadt, Alemania). Finally, an aliquot of each sample was taken for analysis. Reagent blanks were prepared similarly to the samples, and every 10 samples were included in the analytical batch.

For instrumental analyses, an Agilent 7900 ICP-MS (Agilent Technologies, Tokyo, Japan) equipped with standard nickel cones and a crossflow nebulizer with a make-up gas port (×400 nebulizer, Savillex Corporation, MN, USA) was used for all measurements. All data were acquired and processed with Agilent MassHunter data analysis software (version 4.2). ICP-MS was optimized daily using a tuning solution consisting of a mixture of Cs, Co, Li, Mg, Tl and Y (Agilent Technologies, Palo Alto, CA, USA).

The entire procedure was validated prior to its use. Pure standards of all elements were acquired in acid solution (5% HNO_3_, 100 mg/L, CPA Chem, Stara Zagora, Bulgaria). Calibration curves were performed in the range from 0.005 ng/mL to 20 ng/mL for REEs and Mes, in the range from 0.01 ng/mL to 100 ng/mL for the toxic elements in the ATSDR listing, and from 2 ng/mL to 70 ug/mL for the essential elements. The recoveries obtained ranged from 84 to 117%. Linear calibration curves were found for all elements (regression coefficients ≥ 0.995). Instrumental LODs and LOQs were calculated as the concentration of the element that produced a signal three and ten times higher, respectively, than that of the averaged blanks. Sample LOQs were calculated by multiplying the instrumental LOQ by the dilution factor applied to the sample during the digestion procedure (1:10 *v*:*v*).

### 2.3. Dietary Exposure Assessment

In order to assess the dietary intakes of every element from these leafy vegetables, the total consumption of each of the green leafy vegetables by the population of the Canary Islands [41,43] was considered and multiplied by the median values of each element (ng/g fresh weight) in each type of vegetable, and divided by the average body weight of the Canarian population (Equation (1)). Estimates were made for two age groups: adults (>17 years and body weight 68.48 kg) and children (7 to 12 years and body weight 34.48 kg) and two consumption scenarios: high consumers (HC, 97.5th percentile) and average consumers (AC, 50th percentile). The individual intakes obtained for each type of leafy green vegetable were added to assess the overall intake of each element from this food category.

(1) $$EDI=\frac{Median\;Leafy\;vegetable\;consumption\;for\;the\;Canary\;Isalnd\;population\;\left(g\right)*Element\;median\;in\;that\;very\;same\;leafy\;vegetable\;\left(\frac{\eta g}{g}\right)}{Body\;Weight\;\left(kg\right)}$$

EDI = Estimated Daily Intake
Body weight for Canary Islands adults: 68.48 kg [31]
Body weight for Canary Islands children: 34.48 kg [31]

The estimated daily intake (EDI) values of each element for each scenario and age group were evaluated according to the reference values and the risk–benefit ratio was charcaterized.

As dietary reference values (DRV, in the case of essential elements), the population reference intake values (PRI) reported by the European Food Safety Authority (EFSA) were used [50,51]. According to the European standard, the PRI is the equivalent of the Recommended Dietary Allowances (RDA) in the United States, i.e., the level of daily dietary intake of a nutrient that is considered sufficient to meet the needs of 97.5% of healthy individuals in each life stage and sex group. In those elements where no PRI was set by EFSA, the Adequate Intake (AI) value was used as the reference value. The AI is the amount established to be somewhat less than adequate for all members of the population group. For those essential elements where the EDI exceeded the PRI or AI, the Tolerable Upper Daily Intake Level (UL) was also considered. The UL is the maximum total chronic intake level of a nutrient from all sources that is considered unlikely to pose a risk of adverse health effects in humans [50,51,52].

As Toxic Reference Values (TRVs), the Tolerable Daily Intake (TDI) values were used for As, Pb, Cd and Hg [53,54,55,56]. Although, in this work, arsenic speciation was not performed, the EFSA scientific opinion of arsenic in foods was taken as a reference to assess the inorganic arsenic intake from leafy vegetables. According to EFSA, the proportion of inorganic arsenic in vegetables is up to 65% of the total As [56]. It was not possible to perform mercury speciation, but as it has been reported that the percentage of the methyl form in vegetables normally seems to vary between 5% and 30%, a conservative approach was considered for the mercury exposure assessment and 20% of the total mercury in the leafy vegetables was considered to be in the form of methylmercury [55].

In the case of REEs (Ce, Dy, Er, Eu, Ga, Gd, Ho, In, La, Lu, Nb, Nd, Pr, Sm, Ta, Tb, Tm, Y, Yb) and ME (Au, Bi, Ga, In, Nb, Pt, Ta, Th, and V), official TRVs have not been established to date. However, some authors have proposed an allowable daily intake of 61 µg/kg body weight (bw) for rare-earth oxides [22,57]. This proposal was based on human health studies in REE in mining areas and on animal experimental results. The aforementioned value was considered as the TRV in the exposure assessment of these REEs and MEs.

### 2.4. Statistical Analysis

All statistical analyses were performed with GraphPad Prism v9.2 software (GraphPad Software, San Diego, CA, USA). The distribution of the variables included in the study was evaluated using the Kolmogorov–Smirnov test. The concentration of most of the contaminants detected did not follow a normal distribution, so the results are expressed in terms of median and range. For this same reason, nonparametric tests were used to check for statistical differences between groups, as these evaluate the median rather than the mean, which is appropriate given the relatively high number of undetected values in some groups. Homogeneity of variance (homoscedasticity) was previously tested using Levene’s test. The Kruskal–Wallis and Mann–Whitney U tests were used as nonparametric tests for overall and pairwise comparisons, respectively. However, as an additional check, pairwise comparisons were also performed using Student’s t-test after logarithmic transformation of the data. A P-value of less than 0.05 (two-tailed) was considered statistically significant. Data below the LOQ but above the LOD were assigned a random value between these two limits. Data below the LOD were considered undetected. Probability levels of <0.05 (two-tailed) were considered statistically significant.

## 3. Results and Discussion

### 3.1. Essential Elements

Table 1 shows the concentration of essential elements in the sampled leafy vegetable. There are significant differences for all of them (*p* < 0.0001).

Spinach, chard, arugula, lettuce sprouts and watercress are the leafy vegetables with the highest concentration of essential elements. It is worth noting the high concentration of Fe and Zn in spinach and arugula, as well as the high levels of Mn in chard. Romaine lettuce has the lowest concentration of essential elements, highlighting the low concentrations of Se, Mo and Co. Iceberg and romaine lettuce are the leafy vegetables with the lowest concentrations of Fe, Zn and Cu. Spinach, arugula, lamb’s lettuce and chard were those with the highest concentration of Cu. Se was found in higher concentrations in lettuce and spinach sprouts and lower concentrations in watercress and iceberg lettuce. In the case of Mn, it is worth highlighting the high concentrations found in chard, spinach and watercress and the low concentrations found in romaine lettuce and iceberg lettuce. Mo levels are noteworthy in lamb’s lettuce, arugula and watercress. Co is more present in arugula and spinach. These results are similar to those in the study by Al Jassir et al. [9], in which Cu and Zn were also found to reach relevant levels in green leafy vegetables.

The dietary exposures to these essential metals from these vegetables are shown in Table 2. Among all the studied leafy vegetables, watercress and spinach are the main dietary sources of essential elements for Canary Islands adults and children.

In adult 50th percentile consumers (average consumers (AC)) the greatest contribution of essential elements comes from arugula, spinach, watercress, chard, and lamb’s lettuce. In this scenario, lamb’s lettuce contributes up to 39% of the DRV of Mo.

In adult high consumers, spinach contributes the most to the essential elements reference values, followed by arugula and watercress. Regarding Fe, spinach and arugula can contribute up to 70% and 46% of the DRV, respectively. There is also a high intake of Mo from arugula (83% of the DRV) and lamb’s lettuce (197% of the DRV). The contribution of Co from spinach is 72% of the DRV, and from arugula this is 59% of the DRV.

In children, the contribution of Mo to the reference intake values is noteworthy because of the consumption of lamb’s lettuce (40% in AC; 201.9% in HC) and watercress (21% in AC; 104% in HC). These results confirm the importance of monitoring the dietary intake of Mo from this and other dietary sources to assess the derived risks [58]. Furthermore, in the case of children who are high consumers of leafy vegetables, while the contribution of Fe from the consumption of spinach reaches 77% of the DRV, the contribution of the consumption of watercress contributes 26% of the DRV. This situation, observed for Fe, is also observed for the rest of the essential elements, except for the case of Se in watercress. Spinach is also identified as an important source of Co (78% of the DRV) and Mn (77% of the DRV) in children who are high consumers of leafy vegetables. Similar results for spinach have previously been reported by Ellen et al. [59]. These authors pointed to this vegetable being a relevant dietary source of Cu, Mn and Zn, since its consumption provides more than 10% of the DRV of these three essential metals.

**Figure 2:**
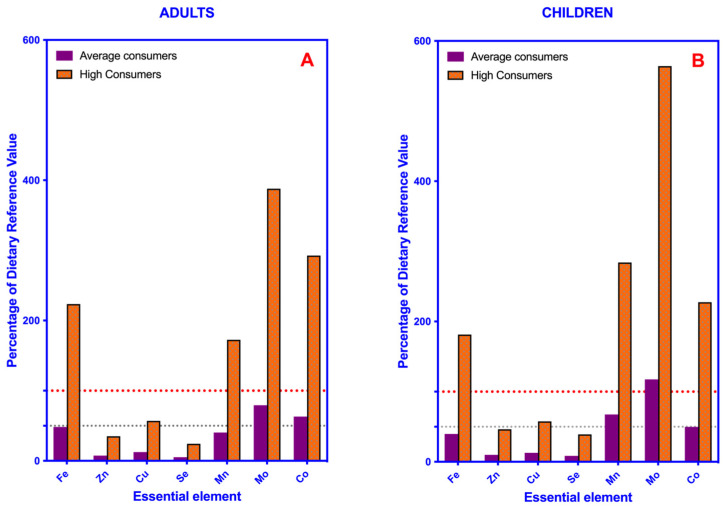
Bar plot showing the percentage of the dietary reference values (DRV) of essential elements provided by the consumption of leafy green vegetables in average and high consumers. (**A**) Adults. (**B**) Children. Red and gray dotted lines indicate 100% and 50% of the DRV of each element, respectively.

Figure 2 shows the exposure to each element from the daily consumption of leafy green vegetables in adults and children and for the two consumption scenarios considered (AC and HC) and evaluates the percentage contribution of the daily intake of green vegetables to the DRV (the dashed line represents 100% of the DVR). Fe, Mn, Mo and Co are the elements from which the consumption of leafy vegetables contributes the most to the DRV in high consumers (HC), in both children and adults, exceeding the DRV in all cases. For the rest of the elements, such as Se, Cu and Zn, the contributions of leafy vegetable consumption to the DRVs are considerable. In the case of the population with an average consumption of leafy vegetables (AC), Mo contributes the most to the diet, exceeding the DRV in the case of children (AC).

### 3.2. Toxic Elements

Table 3 shows the concentration of the detected toxic elements (As, Cd, Hg and Pb) in the different vegetables. Cd is the element that appears in the highest concentration, followed by As and Pb. As suspected, Hg is the toxic metal with the lowest concentrations in vegetables. Spinach is the most contaminated leafy vegetable with these toxic metals and significant differences (p < 0.0001) were detected. Environmental problems from metal and pesticide contamination, their synergistic and antagonistic interactions, as well as their combined toxic effects, have been reported by Alengebawy et al. [46].

While the highest levels of Cd were detected in spinach, romaine lettuce and chard, the lowest levels of Cd were found in lamb’s lettuce. These results are like those previously published by Al Jasir et al. [9] and AESAN [60], in which Cd contamination in these leafy vegetables reaches levels between 10 and 100 µg/kg and the results of the study by Rubio et al. 2006 [16], where Cd was found at levels of 13.62 μg/kg in vegetables. However, the results here are lower than those reported by Mohamed et al. [27], who observed a concentration of 4.13 μg/g of Cd in spinach.

The concentration of Hg and As is low in all the analyzed vegetables, with the highest being Hg in romaine lettuce and sprouts (2.7 ng/g) and As in lamb’s lettuce (18.9 ng/g) and spinach (8.0 ng/g). This situation is similar to that reported by EFSA [55], where the highest Hg concentration was observed in fish and shellfish, and to the study conducted by Martí-Cid et al [61]. In the study by Rubio et al [62], Hg levels in vegetables consumed in the Canary Islands were not detectable.

Arugula and spinach are the most contaminated with Pb. These results are lower than those obtained in the studies conducted by Guerra et al. [13], who reported high levels of Pb in vegetables, and those carried out by EFSA [53]. In contrast to the above, the results of the present study are higher than those found in the study by Rubio et al., 2005 [17], where Pb was detected at a level of 0.14 μg/kg in vegetables.

Table 4 shows the exposure assessment (for adults and children) of potentially toxic elements (PTE) from the consumption of different leafy vegetables produced and consumed in the Canary Islands using the median values of each element and the daily consumption of each vegetable, for high consumers (HC) (percentile 97.5th) and average consumers (AC) (percentile 50th). Lead, cadmium, mercury and arsenic intakes from the consumption of leafy vegetables in the Canary Islands do not show significant values, so the consumption of these vegetables does not pose a health risk. It can be concluded that, in the scenario of average consumption of leafy vegetables, Canary adults have insignificant intakes of toxic metals.

For both adults and children, the average consumption of green leafy vegetables does not generate EDI values that could be considered significant contributions to the established TRVs. However, the high consumption of these leafy vegetables generates relevant EDIs. In the case of HC adults, spinach consumption contributed the highest reference values of toxic elements (7.7% of the TRV of iAs), followed by romaine lettuce (7% of the TRV of MeHg and 2.9% of the TRV of Cd) and watercress (6.7% of the TRV of iAs). In children exposed to a scenario of high consumption of leafy vegetables, the same trend is observed as in adults. Spinach contributes the most to the toxic elements’ dietary exposure (8.3% of the TRV of iAs), followed by romaine lettuce (5% of the TRV of MeHg and 2.4% of the TRV of Cd) and watercress (7.3% of the TRV of iAs).

These results are similar to those reported by Zhu Huan et al. [10], in which the intake of As, Cd, Hg and Pb from vegetables poses a low risk to health but suggests that Cd should be taken into account as a relevant contaminant in vegetables. This same consideration was supported by the risk assessment of Cd exposure conducted by the Spanish Agency for Food Safety and Nutrition (AESAN) [60].

**Figure 3:**
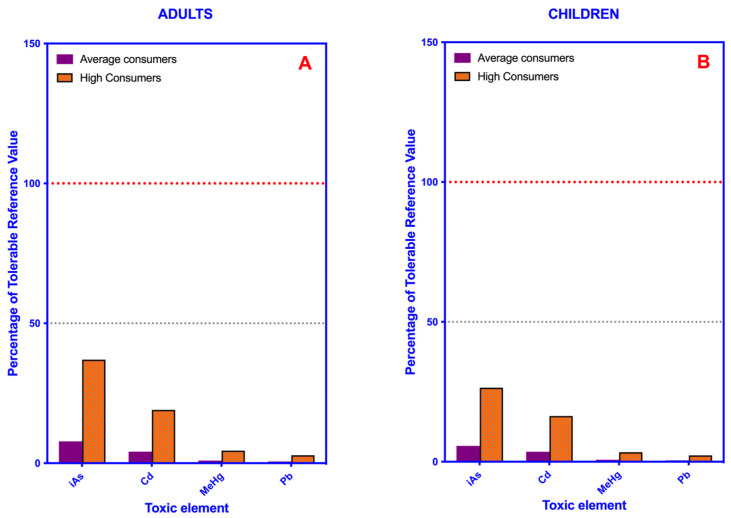
Bar plot showing the percentage of the tolerable daily intake values (TDI) or provisional tolerable weekly intake values (PTWI) of highly toxic elements provided by the consumption of leafy green vegetables in average and high consumers. (**A**) Adults. (**B**) Children. Red and gray dotted lines indicate 100% and 50% of the Toxic Reference Value of each element, respectively.

As can be seen in Figure 3, the exposure to toxic metals from the consumption of leafy vegetables by the Canary population is much lower than the TRVs set for these toxic elements. For both population groups, the dietary intakes of iAs and Cd are worth noting. The rest of the elements do not appear in prominent concentrations.

### 3.3. Potentially Toxic Elements (PTE)

Table 5 shows the concentrations of potentially toxic elements (PTE) in the different analyzed vegetables including the sum of Rare Earth Elements (Sum REE) and the sum of minority elements (Sum ME). Spinach and arugula are the vegetables with the highest concentrations of these elements, followed by watercress and chard. The most abundant elements from highest to lowest concentrations are Al in spinach, Sr in chard and watercress, and Ba in arugula.

These results are in line with those previously published by Ding Guo Jiang et al. [29]. However, they are different from those reported by Liu et al. [23], since these authors found that the Tl levels in vegetables such as lettuce, chard and pak choy exceeded the maximum permitted level (0.5 mg Tl/kg) of the environmental quality standards for food in Germany. Regarding the data on Al, the results are in line with those previously reported by EFSA [21], in which chard, spinach and lettuce are identified as vegetables with a high Al content.

Table 6 shows the estimated dietary exposure (EDI) of potentially toxic elements, including the sum of Rare Earth Elementss (Sum REE) and the sum of minority elements (Sum ME) from the consumption of different leafy vegetables produced and marketed in the Canary Islands.

The elements from which the consumption of leafy vegetables represents a greater contribution to the TRVs for both adults and children are Tl, Al, Sr, Cr and Ba. It is important to highlight the contribution of SUM REE. The high consumption of leafy vegetables (HC scenario) in adults is worth mentioning for the contributions to the TRVs provided by arugula consumption (10% of the TRV of Tl; 7.9% of the TRV of Al), iceberg lettuce intake (8.6% of the TRV of Tl), lamb’s lettuce ingestion (3% of the TRV of Cr), consumption of watercress (3% of the TRV of Cr; 2.6% of the TRV of Sr) and daily chard intake (3% of the TRV of Cr; 1% of the TRV of Ba). The intake generated by the SUM REE from the daily consumption of arugula reaches 0.5% of the toxic reference value.

In the case of children, the contribution from the daily consumption of spinach stands out in both AC profiles (12% of the TRV of Al; 8.6% of the TRV of Tl; 7.1% of the TRV of Cr) and HC profiles (13% TRV of Al; 8.6% TRV of Tl; 7.7% TRV of Cr). This percentage reaches 4% of the TRV of Al in children in the HC scenario of watercress. The dietary exposure to Ba after the consumption of chard makes a greater contribution to the TRV (0.9%) in children following a diet with an HC of leafy vegetables. The Sr contribution from watercress (3% of the TRV) is greater in children with an HC profile.

A recent work investigated the presence of REEs in vegetables in China and leafy vegetables and reported high concentrations. Nevertheless, the risk to human health from their consumption was estimated to be significantly lower than the acceptable daily intake for elements in this group (70 μg kg^−1^ d^−1^) [22]. In the aforementioned study, spinach was found to generate the highest intake of the SUM REE, but only accounts for 1.2% and 1.4% of the value of 70 μg kg^−1^ d^−1^ in the AC and HC scenarios, respectively.

EFSA 2020 [63] reported the scarce research on the potential toxicity of REEs and concluded that the control of these elements in foods of plant origin is necessary. It has been demonstrated that chronic exposure to some of these elements, even at low doses, could lead to a wide range of adverse health effects, even in the early stages of life, such as neurotoxicity, neurodevelopmental toxicity and hepatic alterations. There have been studies suggesting that some of these elements might negatively affect children’s spatial learning and indirectly affect memory.

**Figure 4:**
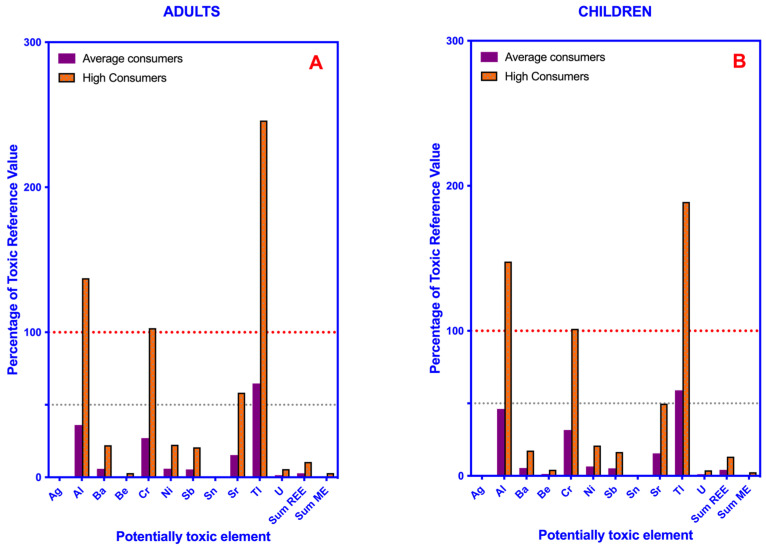
Bar plot showing the percentage of the tolerable daily intake values (TDI) or provisional tolerable weekly intake values (PTWI) of potentially toxic elements, rare-earth elements and minority elements from the consumption of leafy green vegetables in average and high consumers. (**A**) Adults. (**B**) Children. Red and gray dotted lines indicate 100% and 50% of the reference value of each element, respectively.

As can be seen in Figure 4, the consumption of leafy vegetables may involve a worrying exposure to Al, Cr, and especially Tl, in high consumers, in both children or adults.

## 4. Conclusions

The consumption of leafy vegetables provides significant levels of some essential elements (Fe, Mn, Mo, Co and Se), but also of some potentially toxic elements (Al, Cr and Tl), although the levels of toxic elements are insignificant (As, Cd, Pb and Hg). Leafy vegetables with the highest levels of elements are spinach, rucola, watercress and chard. According to the dietary model of the Canary Islands, high consumers of leafy vegetables in this archipelago would meet their daily nutritional requirements of Fe, Mn, Mo and Co, but this population would be exposed to a very moderate risk derived from their exposure to Tl, Al and Cr. Total diet studies on those elements with dietary exposures above the reference values derived from the consumption of this food category, mainly Tl, are recommended.

## Figures and Tables

**Table 1:** Concentrations (ng/g as median and interquartile range (P25–P75) of essential elements in several types of leafy vegetables.

Element	Romaine Lettuce	Iceberg Lettuce	Baby Lettuce	Arugula (Rocket)	Spinach	Lamb’s Lettuce	Watercress	Swiss Chard
Fe	7456 (4105–21,063)	2830 (2229–3448)	13,052 (9957–25,637)	30,037 (7466–38,580)	38,743 (13,124–76,100)	12,578 (10,354–15,465)	12,777 6849–24,522)	18,312 (10,421–30,064)
Zn	1890 (1234–2386)	1725 (1025–2041)	2454 (1964–3092)	3014 (1637–5607)	3748 (2535–9546)	2906 (1953–4421)	3733 (1969–5125)	2704 (2250–3268)
Cu	287.0 (196.9–523.6)	183.5 (128.9–308.5)	287.6 (211.0–399.0)	641.3 (175.4–1066)	698.1 (577.9–1328)	710.1 (417.3–908.0)	490.5 (204.5–739.0)	668.6 (557.3–887.8)
Se	9.3 (6.2–15.2)	5.0 (3.3–14.2)	18.5 (14.7–23.3)	11.4 (6.2–18.8)	17.0 (10.9–43.1)	8.0 (3.8–11.0)	5.3 (2.1–12.4)	11.0 (6.7–16.7)
Mn	1688.3 (981.3–3658.1)	1348.0 (1025.1–1774.3)	2486.1 (1949.8–3181.7)	2793.8 (1401.9–3166.3)	5313.0 (2607.4–8068.2)	4044.0 (2723.8–5055.2)	3491.7 (2542.1–5615.8)	8971.1 (3741.3–12,918.1)
Mo	17.2 (6.8–56.8)	9.8 (3.1–21.9)	14.2 (5.8–18.2)	318.9 (24.5–697.7)	110.6 (81.5–160.7)	758.0 (275.8–1137.2)	118.6 (65.7–343.6)	61.6 (38.5–89.1)
Co	6.2 (3.8–10.6)	4.8 (1.6–13.6)	15.0 (11.5–19.4)	28.8 (8.1–48.1)	29.5 (16.4–57.9)	18.9 (12.8–24.3)	13.8 (7.3–27.2)	17.9 (11.3–26.5)

**Table 2:** Estimated daily intake (EDI) (μg/kg bw/day calculated from the median values) of essential elements from the consumption of leafy vegetables in Canarian adults and children and two consumption scenarios (average and high consumers).

**Adults (>17 y.o.)—68.48 kg bw—Both Genders** **EDI (µg/kg bw/Day) ^a^**																	
**Element**	**Dietary Reference Value ^b^**	**Romaine Lettuce**		**Iceberg Lettuce**		**Baby Lettuce**		**Arugula (Rocket)**		**Spinach**		**Lamb’s Lettuce**		**Watercress**		**Swiss Chard**	
		AC	HC	AC	HC	AC	HC	AC	HC	AC	HC	AC	HC	AC	HC	AC	HC
Fe	160.63 ^c^	3.652	18.420	1.386	6.993	6.393	32.250	14.710	74.220	23.530	112.600	6.161	31.080	7.761	37.890	10.980	29.810
Zn	175.23 ^c^	0.926	4.670	0.845	4.263	1.202	6.064	1.476	7.447	2.276	10.890	1.423	7.180	2.267	11.070	1.622	4.402
Cu	18.98 ^c^	0.141	0.709	0.090	0.453	0.141	0.711	0.314	1.585	0.424	2.029	0.348	1.755	0.298	1.454	0.401	1.088
Se	1.02 ^c^	0.005	0.023	0.002	0.012	0.009	0.046	0.006	0.028	0.010	0.050	0.004	0.020	0.003	0.016	0.007	0.018
Mn	43.81 ^c^	0.827	4.171	0.660	3.331	1.218	6.142	1.369	6.904	3.227	15.440	1.981	9.991	2.121	10.360	5.380	14.600
Mo	0.95 ^c^	0.008	0.042	0.005	0.024	0.007	0.035	0.156	0.788	0.067	0.322	0.371	1.873	0.072	0.352	0.037	0.100
Co	0.12 ^c^	0.003	0.015	0.002	0.012	0.007	0.037	0.014	0.071	0.018	0.086	0.009	0.047	0.008	0.041	0.011	0.029
	**Children (7–12 y.o.)—34.48 kg bw—Both Genders** **EDI (µg/kg bw/Day) ^a^**																
**Element**	**Dietary Reference Value ^b^**	**Romaine Lettuce**		**Iceberg Lettuce**		**Baby Lettuce**		**Arugula (Rocket)**		**Spinach**		**Lamb’s Lettuce**		**Watercress**		**Swiss Chard**	
		AC	HC	AC	HC	AC	HC	AC	HC	AC	HC	AC	HC	AC	HC	AC	HC
Fe	160.63 ^c^	2.915	14.700	1.106	5.581	5.103	25.740	5.872	29.620	25.820	123.600	2.459	12.400	8.434	41.500	9.639	25.710
Zn	108.06 ^c^	0.739	3.727	0.674	3.402	0.960	4.840	0.589	2.972	2.498	11.960	0.568	2.866	2.464	12.130	1.424	3.796
Cu	14.60 ^c^	0.112	0.566	0.072	0.362	0.112	0.567	0.125	0.632	0.465	2.227	0.139	0.700	0.324	1.593	0.352	0.939
Se	0.51 ^c^	0.004	0.018	0.002	0.010	0.007	0.037	0.002	0.011	0.011	0.054	0.002	0.008	0.003	0.017	0.006	0.015
Mn	21.90 ^c^	0.660	3.329	0.527	2.658	0.972	4.902	0.546	2.755	3.541	16.950	0.790	3.987	2.305	11.340	4.722	12.590
Mo	0.37 ^c^	0.007	0.034	0.004	0.019	0.006	0.028	0.062	0.314	0.074	0.353	0.148	0.747	0.078	0.385	0.032	0.086
Co	0.12 ^c^	0.002	0.012	0.002	0.010	0.006	0.030	0.006	0.028	0.020	0.094	0.004	0.019	0.009	0.045	0.009	0.025

^a^ This was calculated considering the consumption (in g/day) of each of the vegetables for the P50 of the children and adult Canary population reported by the Nutritional Survey of the Canary Islands [41]. ^b^ For comparative purposes, the DRVs are expressed in µg/kg bw/day. When a range of different values for males and females is given, the average value was considered. ^c^ Dietary Reference Value (DRV) EFSA [50,51].

**Table 3:** Concentrations (ng/g) as median and interquartile range (P25–P75) of toxic elements in several types of leafy vegetables.

Element	Romaine Lettuce	Iceberg Lettuce	Baby Lettuce	Arugula (Rocket)	Spinach	Lamb’s Lettuce	Watercress	Swiss Chard	*p*
As	1.6 (0.8–6.8)	0.5 (0.4–2.8)	3.9 (2.2–5.1)	7.2 (1.8–8.9)	8.0 (2.9–18.2)	18.9 (8.1–26.2)	6.7 (2.7–16.5)	4.9 (2.6–7.4)	<0.0001
Cd	11.9 (5.3–22.6)	4.0 (1.7–8.6)	9.2 (2.5–36.6)	9.3 (4.3–16.7)	21.5 (11.9–39.7)	1.1 (0.9–2.9)	1.9 (1.1–5.1)	10.5 (4.7–17.9)	<0.0001
Hg	2.7 (2.0–3.6)	2.3 (0.7–2.9)	2.7 (1.5–4.3)	2.3 (1.9–5.4)	1.7 (1.0–2.8)	2.2 (1.4–3.0)	1.0 (0.5–2.9)	1.4 (1.0–3.9)	<0.0001
Pb	4.9 (1.9–10.6)	2.0 (1.2–12.2)	6.9 (3.4–9.5)	13.1 (10.0–18.9)	12.8 (6.0–31.7)	6.4 (4.6–8.2)	7.5 (3.3–14.7)	9.3 (5.8–16.4)	<0.0001

**Table 4:** Estimated daily intake (EDI) (μg/kg bw/day calculated from the median values) of toxic elements from the consumption of leafy vegetables in Canarian adults and children and two consumption scenarios (average and high consumers).

**Adults (>17 y.o.)—68.48 kg bw—Both Genders** **EDI (µg/kg bw/Day) ^a^**																	
**Element**	**Toxic Reference Value ^b^**	**Romaine Lettuce**		**Iceberg Lettuce**		**Baby Lettuce**		**Arugula (Rocket)**		**Spinach**		**Lamb’s Lettuce**		**Watercress**		**Swiss Chard**	
		AC	HC	AC	HC	AC	HC	AC	HC	AC	HC	AC	HC	AC	HC	AC	HC
iAs c	0.30	0.001	0.004	0.000	0.001	0.002	0.010	0.004	0.018	0.005	0.023	0.009	0.044	0.004	0.020	0.003	0.011
Cd	1.00	0.006	0.029	0.002	0.010	0.004	0.023	0.005	0.023	0.013	0.062	0.001	0.003	0.001	0.006	0.006	0.021
MeHg ^d^	0.10	0.001	0.007	0.001	0.006	0.001	0.007	0.001	0.006	0.001	0.005	0.001	0.005	0.001	0.003	0.001	0.004
Pb	6.00	0.002	0.012	0.001	0.005	0.003	0.017	0.006	0.032	0.008	0.037	0.003	0.016	0.005	0.022	0.006	0.022
**Children (7–12 y.o.)—34.48 kg bw—Both Genders** **EDI (µg/kg bw/Day) ^a^**																	
**Element**	**Toxic Reference Value ^a^**	**Romaine Lettuce**		**Iceberg Lettuce**		**Baby Lettuce**		**Arugula (Rocket)**		**Spinach**		**Lamb’s Lettuce**		**Watercress**		**Swiss Chard**	
		AC	HC	AC	HC	AC	HC	AC	HC	AC	HC	AC	HC	AC	HC	AC	HC
iAs ^c^	0.30	0.001	0.003	0.000	0.001	0.002	0.008	0.001	0.007	0.005	0.025	0.004	0.018	0.004	0.022	0.003	0.009
Cd	1.00	0.005	0.024	0.002	0.008	0.004	0.018	0.002	0.009	0.014	0.068	0.000	0.001	0.001	0.006	0.006	0.019
MeHg ^d^	0.10	0.001	0.005	0.001	0.004	0.001	0.005	0.000	0.002	0.001	0.005	0.000	0.002	0.001	0.003	0.001	0.003
Pb	6.00	0.002	0.010	0.001	0.004	0.003	0.014	0.003	0.013	0.009	0.041	0.001	0.006	0.005	0.024	0.005	0.017

^a^ The EDI was calculated considering the consumption (in g/day) of each of the vegetables for the P50 of the Canary population in both adults and children according to the data in the Nutritional Survey of the Canary Islands [41]. ^b^ For comparative purposes, the TRVs are expressed in µg/kg bw/day. When a range of different values for males and females is given, the EFSA average value was considered [53,54]. ^c^ As a reference to calculate the inorganic arsenic exposure, the EFSA scientific opinion of arsenic in foods [59] was considered. According to this opinion, the proportion of inorganic arsenic in vegetables ranges up to 65% [56]. ^d^ It has been established that the percentage of the methyl form in vegetables normally seems to vary between 5% and 30% [55]. With the aim of a conservative approach, the EDI of MeHg was calculated considering that 20% of the total mercury in the leafy vegetables is in the form of methylmercury.

**Table 5:** Concentrations (ng/g as median and interquartile range (P25–P75)) of potentially toxic elements in several types of leafy vegetables.

Element	Romaine Lettuce	Iceberg Lettuce	Baby Lettuce	Arugula (Rocket)	Spinach	Lamb’s Lettuce	Watercress	Swiss Chard	*p*
Ag	0.24 (0.16–0.32)	0.17 (0.12–0.37)	0.16 (0.12–0.33)	0.37 (0.24–0.58)	0.94 (0.48–2.2)	0.28 (0.22–0.34)	0.17 (0.10–0.38)	0.63 (0.42–1.5)	<0.0001
Al	8831.2 (4413.9–26,221.8)	1580.9 (1113.3–5807.3)	13,671.2 (7967.4–20,439.7)	32,145.2 (10,361.8–45,383.9)	41,946.8 11,517.1–83,360.8)	14,185.0 6374.3–23,873.0)	11,810.9 (5583.1–29,424.7)	17,866.4 (9726.5–31,310.9)	<0.0001
Au	1.6 (0.9–2.7)	1.9 (0.8–2.3)	2.1 (0.3–3.4)	1.5 (0.5–3.2)	0.4 (0.3–0.5)	1.4 (0.8–1.9)	0.4 (0.2–1.1)	0.4 (0.2–0.9)	<0.0001
Ba	253.9 (130.5–364.6)	299.0 (181.9–423.2)	431.8 (182.9–1098.3)	687.5 (293.0–1109.4)	511.2 (219.1–935.5)	490.0 (399.6–628.4)	198.3 (71.9–336.1)	1246.1 (334.3–2947.1)	<0.0001
Be	0.40 (0.0–1.17)	0.0 (0.0–0.56)	0.0 (0.0–0.93)	0.23 (0.0–1.03)	2.85 (0.92–4.92)	1.24 (0.91–1.56)	0.91 (0.41–1.81)	1.42 (0.69–2.69)	<0.0001
Cr	21.4 (10.7–37.1)	8.9 (7.2–20.3)	22.8 (16.4–45.5)	52.3 (13.6–176.9)	72.4 (29.5–133.2)	38.0 (28.9–82.3)	31.8 (14.8–61.6)	53.7 (29.1–66.4)	<0.0001
Ni	39.4 (19.9–58.1)	60.4 (13.9–189.4)	49.1 (31.3–124.1)	59.3 (25.9–134.6)	85.7 (22.7–242.1)	40.4 (35.2–46.2)	33.3 (18.1–65.9)	56.5 (29.8–94.0)	0.0154
Sb	0.47 (0.26–0.88)	0.68 (0.23–1.46)	0.78 (0.51–16.08)	0.91 (0.42–1.38)	0.86 (0.68–2.72)	1.02 (0.88–1.22)	1.06 (0.69–1.89)	0.86 (0.54–1.47)	0.0015
Sn	4.4 (1.4–11.1)	1.1 (0.7–6.8)	3.3 (1.6–9.7)	27.9 (18.0–37.2)	13.1 (2.6–13.1)	1.7 (1.4–2.4)	2.7 (0.8–8.2)	3.4 (2.2–9.5)	<0.0001
Sr	1635.1 (1214.9–2763.2)	1917.1 (1068.7–3345.3)	1540.9 (1127.4–10,787.2)	4355.1 (1343.9–8047.4)	4535 (2935.8–8392.6)	3964.5 (3166.7–5525.5)	5324.3 (3262.8–14,408.2)	6510.7 (2833.9–9437.4)	<0.0001
Th	0.6 (0.3–4.6)	0.1 (0.0–0.3)	2.3 (1.2–3.2)	3.4 (0.6–6.4)	6.4 (1.9–14.1)	2.2 (1.0–3.7)	1.8 (0.6–3.9)	2.6 (1.4–4.6)	<0.0001
Tl	1.8 (1.3–2.8)	2.2 (1.0–2.7)	2.1 (0.8–3.1)	2.8 (1.7–5.4)	1.9 (1.2–2.8)	2.2 (1.6–2.6)	0.9 (0.6–1.9)	0.8 (0.6–1.3)	<0.0001
U	0.3 (0.1–1.1)	0.1 (0.0–1.1)	0.9 (0.6–1.3)	1.8 (0.4–2.3)	2.1 (1.4–3.7)	7.9 (2.1–12.6)	1.1 (0.6–2.7)	1.6 (0.7–2.8)	<0.0001
V	10.8 (5.2–41.7)	2.2 (1.6–9.7)	25.8 (19.9–50.0)	54.7 (12.4–70.7)	85.9 (24.5–154.0)	53.1 (19.9–91.9)	30.6 (15.6–61.2)	51.8 (24.1–82.1)	<0.0001
Sum REE ^a^	20.6 (9.1–122.6)	4.0 (3.1–16.1)	81.2 (49.4–134.8)	132.5 (34.4–153.4)	259.5 (53.1–580.2)	62.9 (24.8–85.7)	53.4 (25.8–113.9)	110.8 (54.6–171.1)	<0.0001
Sum ME ^b^	5.9 (3.7–35.3)	1.7 (1.3–3.5)	17.3 (11.4–24.0)	41.1 (10.7–50.4)	62.6 (17.9–112.2)	23.7 85.3–36.9)	17.4 (7.8–31.5)	27.0 (12.8–43.9)	<0.0001

^a^ This is the sum of the individual concentrations of Ce, Dy, Er, Eu, Ga, Gd, Ho, In, La, Lu, Nb, Nd, Pr, Sm, Ta, Tb, Tm, Y and Yb. ^b^ This is the sum of the individual concentrations of Au, Bi, Ga, In, Nb, Pt, Ta, Th, and V.

**Table 6:** Estimated daily intake (EDI) (μg/kg bw/day calculated from the median values) of potentially toxic elements from the consumption of the several types of leafy vegetables in Canarian adults and children and two consumption scenarios (average and high consumers).

**Adults (>17 y.o.)—68.48 kg bw—Both Genders** **EDI (µg/kg bw/Day) ^a^**																	
**Element**	**Toxic Reference Value ^b^**	**Romaine Lettuce**		**Iceberg Lettuce**		**Baby Lettuce**		**Arugula (Rocket)**		**Spinach**		**Lamb’s Lettuce**		**Watercress**		**Swiss Chard**	
		AC	HC	AC	HC	AC	HC	AC	HC	AC	HC	AC	HC	AC	HC	AC	HC
Ag	5	<LOD	0.001	<LOD	<LOD	<LOD	<LOD	<LOD	0.001	0.001	0.001	<LOD	0.001	<LOD	0.001	<LOD	0.001
Al	1000	4.325	21.820	0.774	3.906	6.696	33.780	15.750	79.430	25.480	27960	6.948	35.050	7.174	35.020	10.710	29.080
Ba	200	0.124	0.627	0.146	0.739	0.212	1.067	0.337	1.699	0.311	0.341	0.240	1.211	0.121	0.588	0.747	2.029
Be	2	<LOD	0.001	<LOD	<LOD	<LOD	<LOD	<LOD	0.001	0.002	0.002	0.001	0.003	0.001	0.003	0.001	0.002
Cr	3	0.010	0.053	0.004	0.022	0.011	0.056	0.026	0.130	0.044	0.048	0.019	0.094	0.019	0.094	0.032	0.087
Ni	20	0.019	0.097	0.030	0.149	0.024	0.121	0.029	0.146	0.052	0.057	0.020	0.100	0.020	0.099	0.034	0.092
Sb	0.4	<LOD	0.001	<LOD	0.002	<LOD	0.002	<LOD	0.002	0.001	0.001	<LOD	0.003	0.001	0.003	0.001	0.001
Sn	600	0.002	0.011	0.001	0.003	0.002	0.008	0.014	0.069	0.004	0.004	0.001	0.004	0.002	0.008	0.002	0.006
Sr	600	0.801	4.040	0.939	4.736	0.755	3.808	2.133	10.760	2.754	3.022	1.942	9.797	3.234	15.790	3.904	10.600
Tl	0.07	0.001	0.004	0.001	0.006	0.001	0.005	0.001	0.007	0.001	0.001	0.001	0.005	0.001	0.003	<LOD	0.001
U	3	<LOD	0.001	<LOD	<LOD	<LOD	0.002	0.001	0.004	0.001	0.001	0.004	0.019	0.001	0.003	0.001	0.003
Sum REE ^c^	61	0.010	0.051	0.002	0.010	0.040	0.201	0.065	0.327	0.158	0.173	0.031	0.155	0.032	0.159	0.066	0.180
Sum ME ^d^	NA	0.003	0.015	0.001	0.004	0.008	0.043	0.020	0.101	0.020	0.042	0.012	0.059	0.011	0.052	0.016	0.044
	**Children (7–12 y.o.)—34.48 kg bw—Both Genders** **EDI (µg/kg bw/Day) ^a^**																
**Element**	**Toxic Reference Value ^b^**	**Romaine Lettuce**		**Iceberg Lettuce**		**Baby Lettuce**		**Arugula (Rocket)**		**Spinach**		**Lamb’s Lettuce**		**Watercress**		**Swiss Chard**	
		AC	HC	AC	HC	AC	HC	AC	HC	AC	HC	AC	HC	AC	HC	AC	HC
Ag	5	<LOD	<LOD	<LOD	<LOD	<LOD	<LOD	<LOD	<LOD	0.003	0.003	<LOD	<LOD	<LOD	0.001	<LOD	0.001
Al	1000	3.452	17.420	0.618	3.117	5.345	26.960	6.284	31.700	121.900	133.800	2.773	13.990	7.796	38.370	9.404	25.080
Ba	200	0.099	0.501	0.117	0.590	0.169	0.852	0.134	0.678	1.486	1.631	0.096	0.483	0.131	0.644	0.656	1.749
Be	2	<LOD	0.001	<LOD	<LOD	<LOD	<LOD	<LOD	<LOD	0.008	0.009	<LOD	0.001	0.001	0.003	0.001	0.002
Cr	3	0.008	0.042	0.004	0.018	0.009	0.045	0.010	0.052	0.210	0.231	0.007	0.037	0.021	0.103	0.028	0.075
Ni	20	0.015	0.078	0.024	0.119	0.019	0.097	0.012	0.058	0.249	0.273	0.008	0.040	0.022	0.108	0.030	0.079
Sb	0.4	<LOD	0.001	<LOD	0.001	<LOD	0.002	<LOD	0.001	0.002	0.003	<LOD	0.001	0.001	0.003	<LOD	0.001
Sn	600	0.002	0.009	<LOD	0.002	0.001	0.006	0.005	0.028	0.018	0.020	<LOD	0.002	0.002	0.009	0.002	0.005
Sr	600	0.639	3.225	0.749	3.780	0.603	3.039	0.851	4.294	13.180	14.470	0.775	3.910	3.515	17.300	3.427	9.138
Tl	0.07	0.001	0.003	0.001	0.004	0.001	0.004	0.001	0.003	0.006	0.006	<LOD	0.002	0.001	0.003	<LOD	0.001
U	3	<LOD	0.001	<LOD	<LOD	<LOD	0.002	<LOD	0.002	0.006	0.007	0.002	0.008	0.001	0.004	0.001	0.002
Sum REE ^c^	61	0.008	0.041	0.002	0.008	0.032	0.160	0.026	0.131	0.754	0.828	0.012	0.062	0.035	0.174	0.058	0.156
Sum ME ^d^	NA	0.002	0.012	0.001	0.003	0.007	0.034	0.008	0.040	0.008	0.200	0.005	0.023	0.011	0.057	0.014	0.038

^a^ The EDI was calculated considering the consumption (in g/day) of each of the vegetables for the P50 of the Canary population in both adults and children according to the data in the Nutritional Survey of the Canary Islands [41]. ^b^ For comparative purposes, the TRVs are expressed in µg/kg bw/day, established by EFSA [63]. For Sum REE, the TRVs are those set by EPA [57]. ^c^ This is the sum of the individual concentrations of Ce, Dy, Er, Eu, Ga, Gd, Ho, In, La, Lu, Nb, Nd, Pr, Sm, Ta, Tb, Tm, Y and Yb. ^d^ This is the sum of the individual concentrations of Au, Bi, Ga, In, Nb, Pt, Ta, Th, and V.

## Data Availability

These are data available at the University of Las Palmas de Gran Canaria.
